# Dentistry in the Public Schools

**Published:** 1895-08

**Authors:** E. Proctor Holmes

**Affiliations:** Stoughton, Mass.


### DENTISTRY IN THE PUBLIC SCHOOLS.[1]

BY E. PROCTOR HOLMES, D.M.D., STOUGHTON, MASS.

Mr. President and Gentlemen,—I speak on a subject which, though important, and, I trust, worth your attention, does not pertain to anything of a scientific nature.

Nothing adds more to one's personal appearance than a well-cared-for set of natural teeth. On the other hand, nothing detracts more from a face that nature meant to be comely than decayed, unclean teeth.

We often meet people who, conscious of some glaring defect in their dental organization, never address us without putting the hand to the mouth or contracting the orbicularis oris in the attempt to make the deficiency less apparent.

It is necessary for the moral well-being of a community that its people should be taught to care for and to preserve their teeth. Teach a boy to care for his teeth; instil into his mind the fact that this is necessary for his present and future welfare; keep him at it until what he calls a task becomes a pleasure and a habit, and you have started him well on the road to cleanliness, which leads to godliness.

A boy who cleans his teeth regularly will as regularly wash his face and comb his hair. He will try to be neat in his dress, and this will lead to greater self-respect.

Perhaps some one will say, "The place to teach this is at home."

Yes, this is true; but a little extra teaching on this subject at the public schools will emphasize the home training.

A short time since one of my patients said to me,—

"Dr. Holmes, I must tell you. Harold came home from school the other day, and said to me, 'Oh, mother! what do you think? Teacher says we must brush our teeth every day.' 'Good,' said I; 'now see that you do so.' The next day he came running in, and said, 'Teacher says we must brush our teeth *twice* a day.' 'That's better still,' I replied; 'and when your teacher tells you to brush your teeth *three* times a day I shall say that that is even better,' for," said my patient to me, "you know, doctor, that I try to have Harold take care of his teeth, and I tell him over and over again that he must brush them every day, and two or three times a day,

---

[1] Read before the Harvard Odontological Society, January 31, 1895.

and still he forgets; but when his *teacher* tells him to brush his teeth, why, he thinks he *must* then; and I'm so glad the teacher has taken this step."

It is an acknowledged and a deplorable fact that the general public is not educated up to a proper appreciation of dental science, and we often hear it said that the dentist must be the educator,—must gradually elevate his patients to his own level,—and that as the standard of dentistry is raised, in the same ratio will the people rise to an intelligent appreciation of their teeth and of the dentist who cares for them.

There may be here and there a dentist whose patients, as a whole, are so intelligent that he has never felt the need of any special education among the people regarding the care and proper treatment of the teeth, but among the common people there is a woful ignorance regarding the teeth and their importance, and that even among the more intelligent and well-educated people there is a degree of ignorance displayed utterly at variance with their intelligent appreciation of other subjects.

As a lack of knowledge relative to the teeth and their preservation is confined to no particular class of people, how could we better disseminate this knowledge than through the far-reaching influence of the public schools? and as the number of pupils who attend the public schools is larger in the primary department, growing smaller with each succeeding year, in order to do the greatest good to the greatest number this education should begin very early in life, with the idea of making it quite complete by the time the pupil is ready to graduate from the grammar-school, for two reasons: first, a great percentage of the students complete their education with the grammar-school; and, second, in a great many places physiology, or any subject relating thereto, is not taught in the high schools.

I have frequently asked some of my young patients who were attending the high school what they had learned or were learning there about their teeth, and invariably the answer would be, "Oh, not much."

"What!" I would ask, "does not your physiology contain anything relating to the teeth and the care of them?"

"Oh, we studied physiology at the grammar-school, and I've forgotten what little I ever learned about the teeth."

I have but one patient from the high school who, in speaking of her teeth, designates them by name.

A month or so ago I had for a patient a bright young lady teacher from the primary department of one of our public schools.

Before I had completed her work I found that she hardly knew one tooth from another by name, and was quite surprised when, in answer to my query if she taught her scholars anything pertaining to their teeth, she replied, "Oh, yes, doctor; there is something about the care of the teeth in the 'Child's Health Primer,' although it doesn't have *much* to say on the subject,—it treats mostly on the injurious effects of alcoholic drinks and tobacco; but," she added, "I often tell my scholars that they must brush their teeth every day with salt and water."

I have examined the text-books on physiology used in the schools of one of the representative towns not far from this city, and I can truly say that I am not surprised that the majority of those who graduate from our common schools know little or nothing in regard to their teeth.

The "Child's Health Primer," for primary classes, contains one or two illustrations of the teeth, and about a page and a half of useful information. During the eighth year the text-book in hygienic physiology is begun, and is completed during the ninth year. This text-book of hygienic physiology was written by D. F. Lincoln, M.D., who says, "Nearly the whole of this work has been examined by the following gentlemen: Henry P. Walcut, M.D., James J. Putnam, M.D., Thomas Dwight, M.D.," and others. Suffice it to say that it is a pity that these M.D.'s did not examine the work a little more carefully, or that the part of it relating to the teeth was not examined by a D.M.D. or D.D.S.

The book contains about four pages of descriptive matter, and the synopsis covers about one page, relating to the teeth. There are six illustrations, embracing a cut showing the permanent and the temporary teeth in their relative positions, a vertical section of a bicuspid, and the lower jaw in four stages of its existence.

On page 168 is the following statement: "Children get their first set of teeth (the milk teeth) at various periods, beginning from the fourth to the sixth month of life. This set comprises twenty teeth, —viz., eight incisors or cutting teeth, four canines or eye teeth, and eight bicuspids or grinders, with two roots." In the synopsis reference is again made to the "eight bicuspids in the first set." From page 170, same book, I quote the following: "The enamel gets worn off from the crown of the tooth at the top generally by the twentieth or thirtieth year of age."

Now, while I admit that it may make no great difference to the child if he be taught to call his eight temporary molars bicuspids, and that the result may be the same whether he does or does not

*expect* the enamel to be worn from the "top" of the crowns of his teeth some time between his twentieth and thirtieth years, I *do* claim that the possibility of such misstatements existing in a work of this kind shows with what little importance this branch of physiology—or, more properly speaking, dental anatomy—is regarded.

Let us suppose for a moment that this same book of hygienic physiology contained the statement that the radius and ulnar were two bones of the leg, and that their styloid processes were worn off sometimes between the twentieth and thirtieth years, can we not, those of us who were so fortunate as to receive our instruction in anatomy from Thomas Dwight, M.D., seem to see that gentleman raising his hands in holy horror at the possibility of such an error existing in print, while the eight temporary bicuspids and the enamelless "tops" of the teeth are allowed to pass unchallenged. Yet this, from a dentist's stand-point, is an inexcusable error.

How does our government look at this matter? Does it have any requirements, so far as the teeth are concerned, that must be met by its servants?

To those occupying civil positions it has nothing to say. They may be the happy possessors of their natural teeth, glory in the never-aching qualities of an artificial denture, or have no teeth at all,—it is all the same to "Uncle Sam."

But there are those of its servants who, at the time that they become such, must have teeth that reach a certain, though not very clearly defined, standard of excellence. I refer to the officers and enlisted men of our army.

No matter what stage of physical development a man has reached; no matter what his general qualifications may be; unless his *teeth* are up to a certain standard he cannot enter West Point as a student, nor can he enlist in the regular army.

I think the greater number present will agree that this is as it should be,—that the requirements are not too exacting; but I submit to you, is it right, is it consistent, for our government to demand that a man entering its service as a soldier should have teeth in a fair condition, when, up to the hour of his enlistment, he may have heard not one word relating to the care and importance of those organs which have now become so necessary to his well-being?

Gentlemen, as dentists we have rights which our government is bound to respect.

Of the government that demands that we shall pass an examination before its chosen representatives in order that we may serve its people, we have a right to assert that it shall teach its people how to care for their teeth, and the importance of them, so that they may be able to appreciate in some degree the patience, skill, and faithful service of the dentist.

